# High catechin concentrations detected in *Withania somnifera *(ashwagandha) by high performance liquid chromatography analysis

**DOI:** 10.1186/1472-6882-11-65

**Published:** 2011-08-19

**Authors:** Nadia Alam, Monzur Hossain, Md Ibrahim Khalil, Mohammed Moniruzzaman, Siti Amrah Sulaiman, Siew Hua Gan

**Affiliations:** 1 Department of Botany, Rajshahi University, Bangladesh; 2 Department of Pharmacology, School of Medical Sciences, Universiti Sains Malaysia, 16150 Kubang Kerian, Kelantan, Malaysia; 3 Human Genome Centre, School of Medical Sciences, Universiti Sains Malaysia, 16150 Kubang Kerian, Kelantan, Malaysia

**Keywords:** *Withania somnifera*, spectrophotometry, HPLC, polyphenols, antioxidant, free radical scavenging activity

## Abstract

**Background:**

*Withania somnifera *is an important medicinal plant traditionally used in the treatment of many diseases. The present study was carried out to characterize the phenolic acids, flavonoids and 1,1-diphenyl-2-picrylhydrazyl radical (DPPH) scavenging activities in methanolic extracts of *W. somnifera *fruits, roots and leaves (WSFEt, WSREt and WSLEt).

**Methods:**

WSFEt, WSREt and WSLEt was prepared by using 80% aqueous methanol and total polyphenols, flavonoids as well as DPPH radical scavenging activities were determined by spectrophotometric methods and phenolic acid profiles were determined by HPLC methods.

**Results:**

High concentrations of both phenolics and flavonoids were detected in all parts of the plant with the former ranging between 17.80 ± 5.80 and 32.58 ± 3.16 mg/g (dry weight) and the latter ranging between 15.49 ± 1.02 and 31.58 ± 5.07 mg/g. All of the three different plant parts showed strong DPPH radical scavenging activities (59.16 ± 1.20 to 91.84 ± 0.38%). Eight polyphenols (gallic, syringic, benzoic, p-coumaric and vanillic acids as well as catechin, kaempferol and naringenin) have been identified by HPLC in parts of the plant as well. Among all the polyphenols, catechin was detected in the highest concentration (13.01 ± 8.93 to 30.61 ± 11.41 mg/g).

**Conclusion:**

The results indicating that *W. somnifera *is a plant with strong therapeutic properties thus further supporting its traditional claims. All major parts of *W. somnifera *such as the roots, fruits and leaves provide potential benefits for human health because of its high content of polyphenols and antioxidant activities with the leaves containing the highest amounts of polyphenols specially catechin with strong antioxidant properties.

## Background

Ashwagandha [*Withania somnifera *L. Dunal] (Solanaceae) is an important medicinal plant, commonly-used as a domestic remedy for several diseases in India as well as other parts of the world [1]. It is described as an herbal tonic and health food in the famous book of Vedas and is considered as in 'Indian Ginseng' in traditional Indian system of healing [2]. Several recent reports have demonstrated immunomodulator and antitumor effect of *W. somnifera *as well [3]. Moreover, various parts of the plant have been reported to possess antiserotogenic, anticancer and anabolic properties and have shown beneficial effects in the treatment of arthritis, stress and geriatric problems [4]. *W. somnifera *is also made into dietary supplements with good nutritional properties and phytochemicals. Besides being used among the elderly to increase health vitality, a decoction of *W. somnifera *root is also used as nutrient and health restorative agent among postpartum ladies as it was purported to thicken and increase the nutritive value of the breastmilk when given to nursing mothers. The pharmacological effect of the roots of *W. somnifera *is attributed to its active ingredient, *withanolides *[5] which has a wide range of therapeutic applications [6].

There is a great deal of evidence indicating that excessive free radical production and lipid peroxidations are actively-involved in the pathogenesis of a wide number of chronic diseases, including atherosclerosis [7], cardiac and cerebral ischemia [8], neurodegenerative disorders [9], carcinogenesis [10], diabetes [11] and rheumatic disorders [12] and contributes a major role in the ageing process [13]. Plant-derived antioxidants such as vitamin E, vitamin C, polyphenols including phenolic acids, phenolic diterpenes, flavonoids, catechins, procyanidins and anthocyanins are becoming increasingly important as dietary factors [14]. Supplementation with berry juice [15], flavones from skullcap, catechins from green tea, anthocyanins from chokeberry and condensed tannins from fava beans [16] are indices of oxidative stress protectant in rats. Furthermore, the growing interest in the substitution of synthetic food antioxidants by natural chemicals has fostered research on plant sources and the screening of raw materials for identifying new antioxidants. In this regard, polyphenols are being increasingly reported to exhibit antioxidant effects in foods [17]. Various plants have been analysed for the existence of phenolic acids by HPLC [18]. Plant acids are known to have anticarcinogenic activity [19]. and phenolic compounds are believed to be an important part of the general defence mechanism of many plants against infections [20]. Therefore, it is useful to measure the presence of phenolic compounds in natural substances.

Purification of phenolic acids is very difficult not only due to their isomeric similarities but also due to the influence of various effects such as acid-based treatment, temperature and light on their labile structures [21]. The determination of phenolic acids is important both for their characterization and to facilitate more efficient uses of important plant resources [22].

To date, very limited data exists on phenolic compounds reported in *W. somnifera *leaves, roots and fruits as well as their antioxidant effects to support their traditional claims. Therefore, we aimed to undertake this task in the present study as *W. somnifera *is widely-used. If the presence of phenolic and flavonoid compounds present in *W. somnifera *can be confirmed, the plant can be used as a good possible source of antioxidant.

## Methods

### Plant material

The *W. somnifera *plant parts such as roots, fruits and leaves were collected from field grown plants after six months of cultivation in Botanical Garden, Rajshahi University, Bangladesh. The collected parts of medicinal plant were brought into the laboratory, cleaned and air-dried in shade and then grinded to a fine powder.

### Chemicals and reagents

Reagents such as 1,1-diphenyl-2-picrylhydrazyl radical (DPPH) and Folin-Ciocalteu's reactive were purchased from Sigma-Aldrich (St. Louis, USA). Sodium carbonate (Na_2_CO_3_), aluminium chloride (AlCl_3_), sodium nitrite (NaNO_2_) and sodium hydroxide (NaOH) were purchased from Merck (Darmstadt, Germany). All chemicals used were of analytical grades.

### Preparation of Plant Extracts

*W. somnifera *roots, fruits and leaves extract (WSREt, WSFEt and WSLEt) preparation was performed according to a modified method described by Kahkonen et al., [23]. Grinded dry plant materials (500 mg) were weighed into a test tube followed by the addition of a total of 10 ml of 80% aqueous methanol. The suspension was then stirred slightly. The tubes were sonicated for 5 min and centrifugated for another 10 min at 1500 g and the resulting supernatants were collected. The extraction procedure was repeated and the supernatants were combined before being evaporated to a volume of approximately 1 ml. The concentrated extracts were further lyophilized and weighed.

### Total Polyphenols

Phenolic compounds in *W. somnifera *were estimated by a spectrophotometric determination using a modified Folin-Ciocalteu method Singleton et al., [24]. Briefly, 100 μl of sample extracts (1 mg/ml) was mixed with 1 ml of Folin and Ciocalteu's phenol reagent (2 N). After 3 minutes, 1 ml of 10% Na_2_CO_3 _solution was added to the mixture and adjusted to 10 ml with distilled water. The reaction was kept in the dark for 90 min, after which the absorbance was read at 725 nm (T 80 UV/VIS spectrophotometer, ChromoTek GmbH, Germany). Gallic acid was used to calculate the standard curve (20, 40, 60, 80 and 100 μg/ml, r^2 ^= 0.993). Estimation of the phenolic compounds was carried out in triplicates. The results were mean values ± standard deviations and expressed as milligrams of gallic acid equivalents (GAEs) per g of *W. somnifera *dry weight (DW).

### Determination of total flavonoids

The total flavonoid contents of the *W. somnifera *extracts were determined according to the colorimetric assay method developed by Zhishen et al., [25]. Briefly, 1 ml of properly diluted (1 mg/ml) WSREt, WSFEt and WSLEt were mixed with 4 ml of distilled water. At baseline, 0.3 ml of (5% w/v) NaNO_2 _was added. After five minutes, 0.3 ml of (10% w/v) AlCl_3 _was added followed by the addition of 2 ml of NaOH solution (1 M) six minutes later. After that, the volume was immediately made up to 10 ml, with the addition of 2.4 ml of distilled water. The mixture was shaken vigorously and the absorbance of the mixture was read at 510 nm. A calibration curve was prepared using a standard solution of catechin (20, 40, 60, 80 and 100 μg/ml, r^2 ^= 0.996). The results were expressed as mg catechin equivalents (CEQ) per g of *W. somnifera *(DW).

### DPPH free radical-scavenging activity

The antioxidant capacity of the *W. somnifera *was also studied through the evaluation of the free radical-scavenging effect on the DPPH radical. The determination was based on the method proposed by Ferreira et al., [26]. Briefly, 1 ml (1 mg/ml) of WSREt, WSFEt and WSLEt were mixed with 2.7 ml of methanolic solution containing DPPH radicals (0.024 mg/ml). The mixture was vigorously shaken and left to stand for 60 min in the dark (until their absorbance remained unchanged). The reduction of the DPPH radical was determined by measuring the absorbance at 517 nm [27]. The radical-scavenging activity (RSA) was calculated as a percentage of DPPH discolouration using the equation: % RSA = [(A_DPPH_-A_S_)/A_DPPH_] **× **100, where A_S _is the absorbance of the solution when the sample extract has been added at a particular level and A_DPPH _is the absorbance of the DPPH solution.

### High Performance Liquid Chromatography (HPLC)

The HPLC method was based on the method published by Kaškonienė et al., [28]. Analysis of WSREt, WSFEt and WSLEt were performed by employing an HPLC system (Waters 2695, Milford, MA, USA) equipped with a Photodiode Array Detector (Waters 2996, Milford, MA, USA). The HPLC column was a Merck Purospher Star, RP-18e, (125 × 4 mm, 5 μm) fitted with a guard cartridge packed with the same type of stationary phase (Merck, Darmstadt, Germany). The linear 76 gradient was used at a flow rate of 0.5 ml/min with total analytical time of approximately 35 min. The binary mobile phase consisted of a solvent A (ultra pure water with 0.1% of phosphoric acid) and solvent B (pure methanol with 0.1% of phosphoric acid). Elution from the column was achieved with the following gradient: 0 min to 10 min of solvent B, increased from 35% to 55%; 10-25 min of solvent B, increased to 62%; 25-30 min of solvent B, increased to 85% and the final composition was kept constant till 35 min. All solvents used were of HPLC grade quality. The detection wavelength was done between 200 and 450 nm with specific monitoring at 265 nm. The identification of phenolic compounds was performed by comparing the retention time and UV absorption (λ_max_) of each peak of the analytes with the reference standards. Phenolic acids (gallic, syringic, caffeic, vanillic, p-coumaric, benzoic and transcinnamic acids) as well as flavonoids (catechin, naringenin, luteolin, hesperetin, kaempferol, apigenin, naringin) were purchased from Sigma (St. Louis, MO, USA) and were used as reference standards.

### Statistical analysis

All analyses were carried out in triplicates and the data was expressed as means ± standard deviations (SD). The data was analyzed using (Statistical Packages for Social Science 12.0) (SPSS Inc., USA) and MS Excel 2003. One-way analysis of variance (ANOVA) followed by Tukey's honestly significant difference post hoc test was used to compare the phenol contents, FRAP values, DPPH scavenging activities and colour parameters of WSREt, WSFEt and WSLEt (Table 1). The differences between means at 95% (p < 0.05) confidence level were considered statistically significant. Correlations were obtained by Pearson's correlation coefficient (r) in bivariate linear correlations.

**Table 1:** Spectrophotometric analysis of phenolics, flavonoids and antioxidant properties of *W. somnifera *roots, fruits and leaves.

*W. somnifera*	Phenolics mg GAE/g (DW)	Flavonoids mg CEQ/g (DW)	% of DPPH inhibition
Roots	17.80 ± 5.80^c^	15.49 ± 1.02^c^	59.16 ± 1.20^c^
Fruits	22.29 ± 1.99^b^	21.15 ± 5.32^b^	70.38 ± 0.84^b^
Leaves	32.58 ± 3.16^a^	31.58 ± 5.07^a^	91.84 ± 0.38^a^

In each column, values with different letters (superscripts) indicate significant differences (p < 0.05). DW = dry weight

## Results

### Phenolic content

The contents of total polyphenols (mg GAE/g) of WSREt, WSFEt and WSLEt were investigated using the modified Folin-Ciocalteu assay which is sensitive to phenol and polyphenols entities and other electron donating antioxidants such as ascorbic acid and vitamin E. The sources of the analysed WSREt, WSFEt as well as WSLEt were significantly different (p < 0.05), as shown in (Table 1). Among the three different *W. somnifera *extracts, the concentrations of polyphenols was found to be lowest in WSREt (17.80 ± 5.80 mg/g) and highest in WSLEt (32.58 ± 3.16 mg/g).

### Flavonoids content

The total contents of flavonoids of the three different *W. somnifera *extracts were also determined. Flavonoids were detected in high concentrations ranging from 15.49 ± 1.02 (WSREt) to 31.58 ± 5.07 (WSLEt) mg CEQ/g (Table 1).

### DPPH radical scavenging activity

There were significant differences in terms of their scavenging abilities present among the WSREt, WSFEt as well as WSLEt samples, expressed as percentage of inhibition on the DPPH radical (Table 1). Among the three extracts, the lowest scavenging activity was found in WSREt (59.16 ± 1.20%) while the highest activity was found in WSLEt (91.84 ± 0.38%). The DPPH radical scavenging test is one of the fastest tests available to investigate the overall hydrogen/electron donating activity of single antioxidants and health-promoting dietary antioxidant supplements. The reasons behind the markedly higher radical scavenging capacity exhibited by the different types of *W. somnifera *extracts probably lie in their diverse botanical origin. Antioxidant potential of *W. somnifera *extracts is directly related to its phenolic and flavonoids content.

### Correlations

The correlations among the phenolic compounds, flavonoids and DPPH radical scavenging activities are shown in (Table 2). The correlation matrix showed that significant linear correlation exists between the results of all three analytical methods employed indicating that the three measurements are reliable indicators of antioxidant activities. The lowest linear correlation value at r = 0.962 (p = 0.01) and the highest correlation value at r = 0.995. Both phenolic compounds and DPPH radical scavenging activity are strongly correlated (r = 0.995 and 0.983 respectively) with DPPH radical scavenging activities. The significant correlations existing between phenolic compounds and DPPH radical scavenging activities indicate the strong antioxidant properties of the tested WSREt, WSFEt and WSLEt. Similar to our findings, some literature also reported strong correlation between the antioxidant capacity and total phenolic contents [29] further suggesting that polyphenols are the major components responsible for the antioxidant effects of WSREt, WSFEt and WSLEt.

**Table 2:** Correlations matrix among phenolics, flavonoids content and free radical scavenging activities Correlations

	phenolics	Flavonoids	DPPH
phenolics	1	0.995(**)	0.983(**)
Flavonoids	0.995(**)	1	0.962(**)
DPPH	0.983(**)	0.962(**)	1

** Correlation is significant at the 0.01 level (2-tailed).

### HPLC analysis

**Figure 1:**
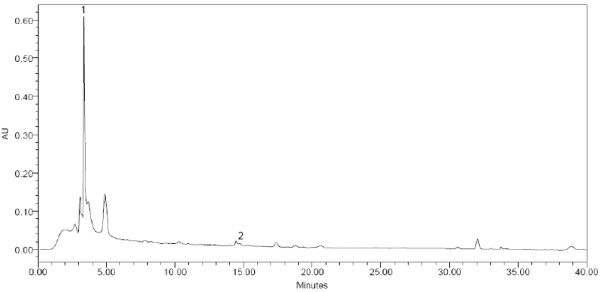
**HPLC chromatogram of *W. somnifera *roots**. (1) catechin and (2) benzoic acid.

**Figure 2:**
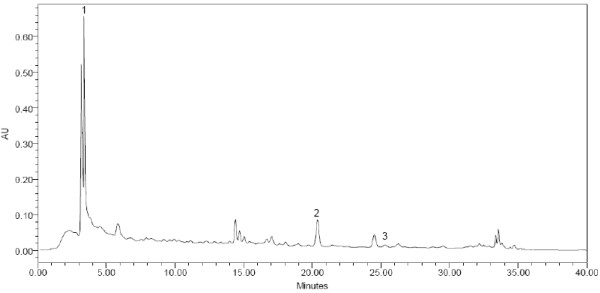
**HPLC chromatogram of *W. somnifera *fruits**. (1) catechin (2) naringenin and (3) kaempferol.

**Figure 3:**
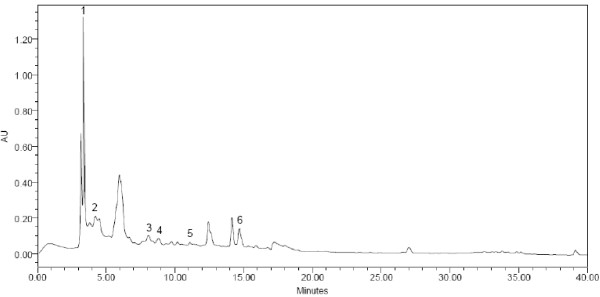
**HPLC chromatogram of *W. somnifera *leaves**. (1) catechin, (2) gallic acid, (3) syringic acid, (4) vanillic acid, (5) p-coumaric acid and (6) benzoic acid.

**Figure 4:**
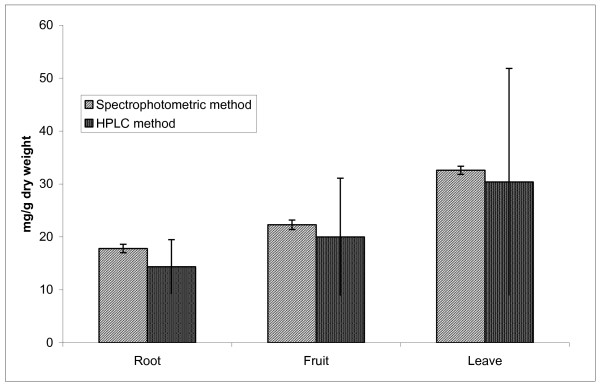
**Comparison of total values of phenolic and flavonoid compounds (mg/g) of *W. somnifera *roots, fruits and leaves obtained by spectrophotometric and HPLC method (p < 0.05)**.

Sixteen phenolic and flavonoid standards were compared with the chromatograms produced by the unknown *W. somnifera *extracts. HPLC analysis of phenolic and flavonoids compounds in WSREt, WSFEt as well as WSLEt showed that only catechin is commonly found in all of the three extracts analyzed. Eight polyphenols (five phenolic acids and three types of flavonoids) have been identified and the phenolics patterns of all plant parts were confirmed to contain gallic, syringic, benzoic, p-coumaric and vanillic acids as well as the flavonoids catechin, kaempferol and naringenin.

(Figures 1, 2, 3) show the HPLC chromatograms obtained from WSREt, WSFEt and WSLEt. Six phenolic compounds were detected in WSLEt whereas, three compounds were identified in WSFEt and only two compounds were identified in WSREt. The unknown compounds that may have had similar flavonoid and phenolic acid spectra and chromatographic behaviours (shown as extra peaks in the figures) were also detected. However, they could not be fully identified due to lack of standard compounds. (Figure 4) compared the total phenolic compounds of sample extracts obtained when using spectrophotometric and HPLC methods. Overall, spectrophotometric methods tend to report higher levels of phenolics when compared to HPLC method.

## Discussion

To our knowledge, our study is the first to identify phenolic compounds present in *W. somnifera*. The present study confirmed the presence of phenolic compounds, flavonoids and antioxidant activities in WSREt, WSFEt and WSLEt. High concentrations of phenolic compounds were found in the different parts of *W. somnifera *with significant variations in the amount. (Table 1) showed high content of phenolics and flavonoids in WSLEt (32.58 ± 3.16 and 31.58 ± 5.07 respectively) while that in WSREt was low (17.80 ± 5.80 and 15.49 ± 1.02 respectively). This indicates that the leaves of *W. somnifera *should be consumed for its antioxidant effects. Several reports have shown that different plant parts have variable polyphenols compositions, as shown by our findings [30].

There is very poor data on analysis of phenolic compounds in *W. somnifera*. Udayakumar et al., [30] from India reported that the presence of total phenolic compounds in WSREt was 28.26 mg/g while that of flavonoids was 17.32 mg/g. For WSLEt it was 5.4 mg/g total phenolic compounds and 5.1 mg/g flavonoids both of which were different from our study perhaps due to the different source of *W. somnifera *and polyphenols of plant parts which may also be related to the colour, maturity and environment. However, the spectrophotometric method tend to overestimate the phenolics content with respect to the chromatographic method perhaps due to the fact that non-phenolic materials present in the investigated extracts interfered in the spectrophotometric analysis [31].

The scavenging ability of DPPH free radical is extensively used to screen the antioxidant potential of naturally-derived foods and plants. Methanol was employed in this study to extract the low molecular weight and moderately polar substances because of its wide solubility properties. We found that WSREt, WSFEt and WSLEt exhibited free radical DPPH scavenging abilities (Table 1). In this study, we attempted to isolate the active compounds responsible for antioxidant activities in WSREt, WSFEt and WSLEt. The antioxidant capacity has been shown to be directly related with the total phenolic content (Table 2) which was in agreement with many previous reports [32-34]. In addition, the flavonoids contributed to almost all of the total phenolic content (Table 1), which indicated that the flavonoids in *W. somnifera *are important constituents responsible for the bioactivities.

In HPLC analysis, six compounds were identified in WSLEt while three were identified in WSFEt and two were identified in WSREt. Out of the eight phenolic compounds catechin was found in the highest concentration compared to others amounting 12.82 mg/g in WSREt, 19.48 in WSFEt and 28.38 mg/g in WSLEt (Table 3). This indicates that WSREt, WSFEt and WSLEt are rich sources of catechin. Catechin is one of the most important polyphenols that provide health benefits and is found in high quantities in green tea which is widely known for its strong antioxidant properties. There are many reports on catechin which described its therapeutic role in human health. Modern studies have found that catechin is responsible for antioxidant activity, anti-ageing properties and cardiac health maintenance [35]. Catechins' beneficial effects are attributed to its ability to reduce oxidative stress, lipid peroxidation, free radical generation and unhealthy low density lipoprotein (LDL) cholesterol-oxidation [36]. There is also an evidence that suggests that catechins have a role in the protection against degenerative disorders [37]. Throughout the experiments, some catechins have also been shown to inhibit a key enzyme (squalene epoxidase) in the pathway of cholesterol biosynthesis [38]. The potent antioxidant properties of catechin reduce free radical damage to cells and prevent the oxidation of LDL cholesterol [39]. Besides catechin, other phenolic compounds found in the WSREt, WSFEt and WSLEt may also contribute to its medicinal and antioxidant properties [40]. Further studies to isolate individual active principles and antioxidant activity of individual extracts of roots, fruits as well as leaves through radical scavenging assay and their pharmacological validation in terms of modern medicine will be of great pharmacological importance in future which is under our consideration.

**Table 3:** Phenolic acids and flavonoids compounds detected in *Withania somnifera *roots, fruits and leaves using high performance liquid chromatography analysis.

SL No	Standard compounds	Retention time	**λ**_**max **_**(nm)**	Quantity of the identified compounds (mg/g DW)		
				
				Roots	Fruits	Leaves
1	Catechin	3.36	278	12.82	19.48	28.38
2	Gallic acid	4.12	269, 216	ND	ND	0.18
3	Syringic acid	8.10	268, 216	ND	ND	0.30
4	Vanillic acid	8.61	224, 249, 269	ND	ND	0.15
5	p-coumaric acid	10.97	264, 286, 310	ND	ND	0.80
6	Benzoic acid	15.33	272, 241	0.19	ND	0.80
7	Naringenin	20.18	277, 292, 308	ND	0.50	ND
8	Kaempferol	25.59	361	ND	0.06	ND
Total phenolic compounds				13.01 ± 8.93	20.04 ± 11.09	30.61 ± 11.41

(ND = Not detected), DW = dry weight

## Conclusion

Five phenolics (gallic, syringic, benzoic, p-coumaric and vanillic acids) and three flavonoids (catechin, kaempferol, and naringenin) have been identified in WSREt, WSFEt and WSLEt and catechin was found in high concentrations especially in the leaves part, confirming the antioxidant potential and health benefits of *W. somnifera.*

## Competing interests

The authors declare that they have no competing interests.

## Authors' contributions

NA, MIK and MM have carried out the experimental parts of this investigation. MH, SAS and SHG supervised the work, evaluated the results and corrected the manuscript for publication. All authors read and approved the final manuscript.

## Pre-publication history

The pre-publication history for this paper can be accessed here:

http://www.biomedcentral.com/1472-6882/11/65/prepub
